# Dislodging Dichromate in Mine Slops Applying Flat Supplying Membrane Equipment Containing Carrier N235/7301

**DOI:** 10.3390/membranes12090880

**Published:** 2022-09-12

**Authors:** Liang Pei

**Affiliations:** 1 National Engineering Technology Research Center for Desert-Oasis Ecological Construction, Xinjiang Institute of Ecology and Geography, Chinese Academy of Sciences, Urumqi 830011, China; peiliang@ms.xjb.ac.cn; 2 Xinjiang Key Laboratory of Environmental Pollution and Bioremediation, Xinjiang Institute of Ecology and Geography, Chinese Academy of Sciences, Urumqi 830011, China; 3 Institute of Geographic Sciences and Natural Resources Research, Chinese Academy of Sciences, Beijing 100101, China; 4 University of Chinese Academy of Sciences, Beijing 100049, China

**Keywords:** mine slops, Tri (octyl decyl) alkyl tertiary amine, dichromate, flat supplying membrane equipment

## Abstract

A novel flat supplying membrane equipment (FSME) with a sodium hydroxide solution and a mixture of N235/7301 and petroleum has been studied for dislodging dichromate (which can be expressed as Cr (VI) or Cr_2_O_7_^2−^) from simulated mine slops. The FSME contained three parts: as a feeding cell, a reacting cell, and a supplying cell. The flat Kynoar membrane was inlaid in the middle of the reacting cell, using the mixed solutions of petroleum and sodium hydroxide, with Tri (octyl decyl) alkyl tertiary amine (N235/7301) as the carrier in the supplying cell and the mine slops with Cr (VI) as the feeding section. The impact parameters of pH and the other ion density in the feeding solutions, the voluminal ratio of petroleum to sodium hydroxide solution and N235/7301 concentration in the supplying solutions were investigated for the obtaining of the optimal technique parameters. It was found that the dislodging rate of Cr (VI) could reach 93.3% in 215 min when the concentration of carrier (N235/7301) was 0.20 mol/L, the voluminal ratio of petroleum and sodium hydroxide in the supplying cell was 1:1, the pH of the feeding section was 4.00, and the Cr (VI) cinit was 3.00 × 10^−4^ mol/L. The practicability and steadiness of FSME were gained through the exploration of Cr (VI) adsorption on the membrane surface.

## 1. Introduction

Chromium is a rare refractory metal with excellent physical, chemical and mechanical properties. Chromium products are widely used in the fields of national economy, national defense construction and high-tech industry [1,2]. They have become indispensable and important raw materials and functional materials in modern society [3,4]. According to the data released by the U.S. Geological Survey in January 2021, the global dichromate resource reserves will be 3.4 million tons in 2020, of which China’s dichromate resource reserves account for about 60%, which has an absolute merit in the world [5,6].

Slops containing dichromate mainly come from the mining, beneficiation, metallurgy, electroplating and glass industries [7,8]. This kind of slop is characterized by low metal ion concentration, and the dichromate belongs to rare and precious metals. If the slop containing dichromate is discharged directly without treatment, it will not only harm the environment, but also waste a lot of resources [9,10,11,12]. At present, a significant amount of research has been carried out on the treatment methods of slops containing dichromate. The common primary treatment methods include the precipitation method, the solvent extraction method, the ion exchange method, and the activated carbon adsorption method [5,13]. However, these methods are difficult to recover the dichromate resources in slops, or the recovery cost is high, and hardly completely extract dichromate [14,15]. Liquid membrane separation technology has the merits of good selectivity, fast extraction speed, simple equipment, and is energy saving [16,17]. At present, it has been studied and applied in the petrochemical industry, hydrometallurgy, medicine, agriculture, and slops treatment [18,19,20,21]. The dislodging of dichromate by the emulsion liquid membrane method has been reported [8,9,10].

Extraction technology and membrane technology are better methods to treat industrial heavy metal wastewater. In particular, membrane technologies have broad application prospects. Some scholars used the extraction method to dislodge the heavy metals and compounds, but the efficiency was very low and the effective use times were less [22,23,24,25]. Recently, some scholars have used the new liquid membrane technology to dislodge heavy metals [26]. However, due to the lack of supplementary solution, the dislodge efficiency was low, the membrane was used less often, and the cost was high [27,28]. Some people also used the hollow fiber liquid membrane device to study the dislodging of molybdenum [29]. It was found that the removal rate could be increased by 10% after the supplementary liquid was used, and the use times of the membrane increased significantly [30]. Some also used the ion-exchange membrane to dislodge heavy metal compounds, and the effect was significantly lower than that of membrane technology [31]. Many studies also showed that the parameter selection of liquid membrane technology is similar to that of extraction technology, but the dislodging efficiency was much higher [32]. Because the organic membrane solution is volatile, the stability of the liquid membrane and the extraction method is also affected [33]. Therefore, whether the liquid membrane technology can operate in a closed manner will be the focus of the research. The volatilization of the organic membrane solution is controlled, which can improve the removal rate and stability [34].

In this work, novel flat supplying membrane equipment (FSME) with mixed N235/7301 dissolved in petroleum and sodium hydroxide solvent was designed based on SLM to efficiently remove Cr (VI) from mine slops with high stability. N235/7301 was used as a carrier. The stability was enhanced and the extraction rate of Cr (VI) was significantly increased by the separation of the reacting cell with the feeding and supplying cells in the novel design. The selection of variable parameters was determined according to the research results of extraction technology and liquid membrane technology [22,23,24,25]. The influencing factors, optimal technique parameters, and stability of the novel FSME were discussed with the expectation of breakthrough industrial applications for the dislodging of Cr (VI) from the mine slops.

## 2. Materials and Methods

### 2.1. The Design of FSME and Reaction Mechanisms

**Figure 1:**
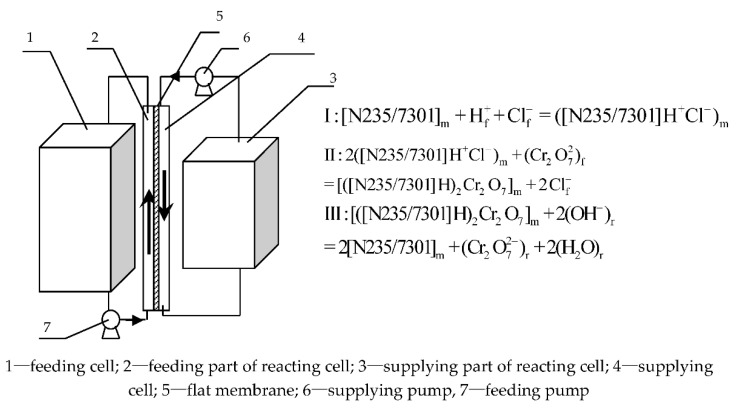
The structure and reaction mechanisms of the flat supplying membrane equipment (FSME) with mixed N235/7301 dissolved in petroleum and sodium hydroxide solvent for the dislodging of Cr (VI) [35].

The framework of FSME and its reaction mechanisms are shown in Figure 1. Basically, FSME contained three cells, namely the feeding cell (400 mL), reacting cell (300 mL), and the enrichment cell (200 mL), connected with the pipeline connecting the two power pumps (such as the supplying pump and feeding pump). The reaction cell was divided into a feeding section (200 mL) and a supplying section (200 mL) by an embedded flat membrane. The equipment first uses a feed pump to introduce Cr (VI) and buffer solution (HAC-NaAc) in the feed tank into the feeding part of the reaction cell. [35]. The certain voluminal ratio of the mixed oil-water solution, included membrane solvent (petroleum) with carrier N235/7301 and stripping solution (sodium hydroxide), in the supplying cell was placed into the supplying part of the reacting cell by the supplying pump for Cr (VI) dislodging and membrane solution supplying simultaneously, thus improving the operational stability of the system and increasing the dislodging efficiency of Cr (VI) [35]. 

The synchronous dislodging process involves various (equilibrium) reactions [36]: (a) the Cr (VI) spreading from the feeding section into the limiting surface of membrane and the feeding liquid; (b) the Cr (VI) dislodging from the feeding liquid with organic N235/7301 in petroleum, expressed as chemical equation I and II in Figure 1, resulted in the metal-complex [([N235/7301]H)_6_Cr_2_O_7_]; (c) the diffusion of metal-complex [([N235/7301]H)_6_Cr_2_O_7_] through the membrane from the limiting surface of membrane and feeding liquid of the feeding section to the limiting surface of the membrane and supplying the liquid of the supplying section in the reaction equipment; (d) [([N235/7301]H)_6_Cr_2_O_7_] decomposed into two substances into N235/7301 and Cr (VI) in the limiting surface membrane and supplying solution, according to chemical equation III in Figure 1, with the sodium hydroxide as stripping agent of Cr (VI) and petroleum as the solvent of N235/7301; (e) the Cr (VI) enrichment of the supplying section; (f) the supply pump re-inputs N235/7301 from the supply cell into the reaction tank. Since N235/731 diffuses through the membrane [35,36], the reaction cell can react with the feed liquid to increase the steadiness of the system.

### 2.2. Materials and Reagent

Flat porous membrane (The material is Kynoar) was used in our new design, with an aperture of 0.28 μm, thickness of film of 75 μm, a twist rate of 1.82, and a porosity of 70%~80% (Shanghai Yadong Nuclear Grade Resin Co., Ltd., Shanghai, China) [35]. Tri (octyl decyl) alkyl tertiary amine (N235/7301) was used as the carrier in this work, with the density of 0.871 and purity of 94.8% (Shanghai laiyashi Chemical Co., Ltd.). HCl and Cr (VI) solution was mixed as a feeding solution to imitate industrial slops containing Cr (VI). The buffer solution (HAc-NaAc) was applied for the pH adjustment (2.6–4.0) of the feeding solution and the mixed solution of KCl and KNO_3_ were applied for the monitoring of the ion-density in the feeding section to imitate original industrial slops. Sodium hydroxide is selected as the resolving liquid and the homemade petroleum is used as the organic solvent. The mixed solutions of petroleum with N235/7301 and sodium hydroxide solution were used as the supplying solution. All the reagents (except petroleum) were of analytical grade.

### 2.3. Test Method

For the methods and conditions of the test and the determination in this study, please refer to our previous relevant studies [35]. To determine the pH value of the solution, we used a digital acidity ion meter (Phs-3c; Shanghai KANGYI Instrument Co., Ltd., Shanghai, China). 4-(2-pyridylazo) resorcinol (standard rod number) was used as the developer. The absorbance was measured at 470 nm by spectrophotometry to determine the molarity of Cr (VI). The symbol *Re* denotes the dislodging efficiency and *Rr* denotes the dislodging rate. The dislodging efficiency (separation coefficient) is calculated as the logarithm of the ratio of the Cr (VI) concentration (*C_t_*) and the initial Cr (VI) concentration (*C*_0_) in the feed tank, as shown in Equation (1), and the removal rate is calculated as Equation (2).

(1) $$Re=-\ln(\frac{C_{t}}{C_{0}})$$

(2) $$Rr=\frac{C_{0}-C_{t}}{C_{0}}\times 100$$

### 2.4. Experimental Procedure

All experiments were accomplished at 20 ± 3 °C with the FSME. The effective area is 50 cm^2^. We set the flow rate of the two pumps at 13.1 mL/min. Before the test, we first immersed the PVDF membrane in the petroleum solution with N235/7301 for about 1 h, then took it out to dry naturally and embedded it in the reaction cell. The prepared supplying and feeding chemical substances were introduced into the feeding solution and the supplying solution separately. Then, with the start of the feeding pump and the supplying pump, the experiment officially began. Samples were taken from the feeding and supplying cells to test the Cr (VI) mor-concentration at 60/120/150/180/215 min, individually. 

## 3. Results and Discussion

### 3.1. Effects of pH and Ion-Density in the Feeding Cell

**Figure 2:**
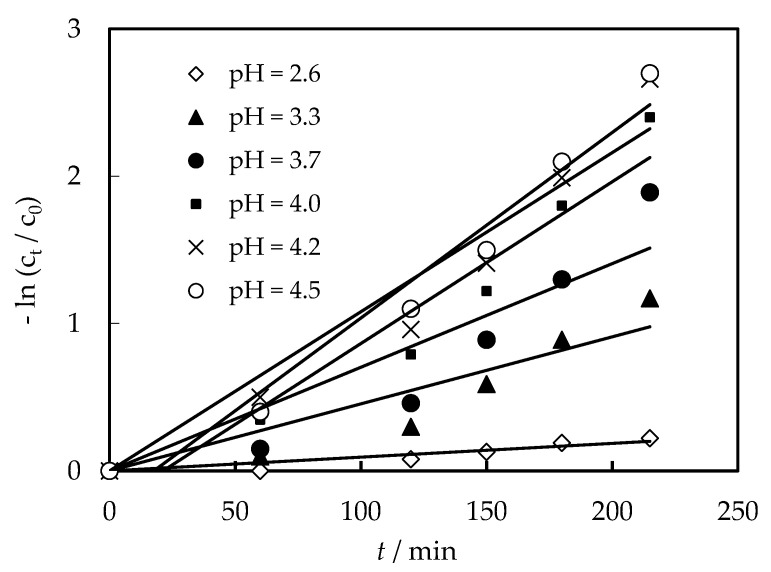
Effects of pH of the feeding solution on the dislodging efficiency of Cr (VI).

Basically, the dislodging power in the experimental equipment comes from the acidity difference between the feed part and the supply part. The pH of the feed solution plays a key role in the dislodging [12]. In order to study the impact of the pH value of the feed solution on the Cr (VI) dislodging efficiency in our work, the initial experimental conditions could be set as: the volumetric proportion of petroleum to sodium hydroxide in the supply cell was constant at 1:1, the molarity of sodium hydroxide liquid was 0.30 mol/L, and the carrier molarity in the mixture liquid in the supply cell was 0.18 mol/L. The initial molarity of Cr (VI) was configured to 3.00 × 10^−4^ mol/L. As shown in Figure 2, when the pH of the feed solution increased from 2.6 to 4.5, the dislodging efficiency gradually increased.

As mentioned above, the driving force for the mass diffusion of complex [([N235/7301]H)_6_Cr_2_O_7_] is pH, that is, when the pH decreases, the removal efficiency of Cr (VI) also decreases. Also, when H^+^ increased, the formation of ([N235/7301]H^+^Cl^−^) (Equation I in Figure 1) concentration, which is benefit for the generation of [([N235/7301]H)_6_Cr_2_O_7_] (Equation 2 in Figure 1). However, in our experiment, we found the formation of WO_2_^2^^−^ when pH decreased, which hindered the reactions of Equation. II and lowered the dislodging efficiency. As a result, the higher the pH was, the more inefficient the dislodging rate was. However, the dislodging efficiency was close when the pH was from 4.0 to 4.5. In the following experiments, a pH of 4.00 was suitable as the optimal pH condition of the feed portion.

**Figure 3:**
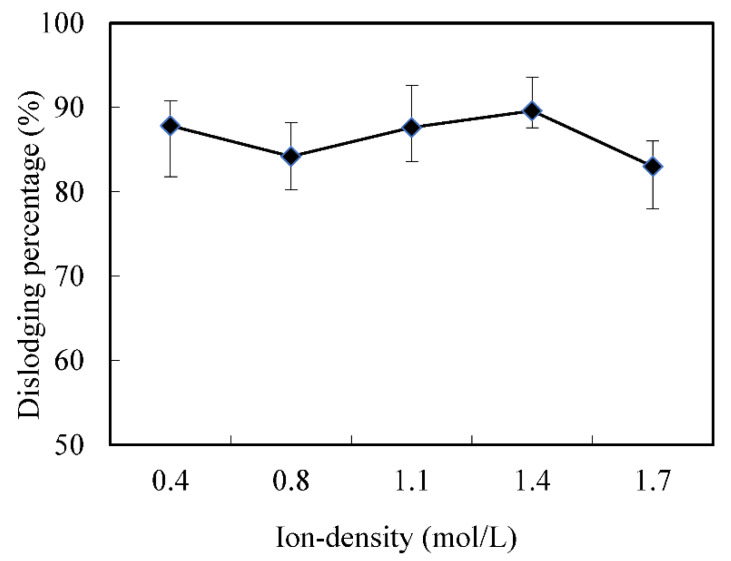
Effects of the ion-density in the feeding cell on the dislodging rate of Cr (VI).

In the industrial slops, there always existed other ions, which may affect the dislodging of Cr (VI). Thus, the NaCl and KNO_3_ were used to simulate the industrial slops, and to investigate the effect of the initial ion-density in the feeding cell on the dislodging rate of Cr (VI). As shown in Figure 3, the dislodging rate of Cr (VI) increased when the initial ion-density changed from 0.4 mol/L to 1.7 mol/L and was kept higher than 80%. This result shows that during the removal process, other ions in the feed cell have little effect on the efficiency of the technical system, which is consistent with previous research results [14,23].

### 3.2. Effects of the Voluminal Ratio and Carrier Concentration in the Supplying Cell

**Figure 4:**
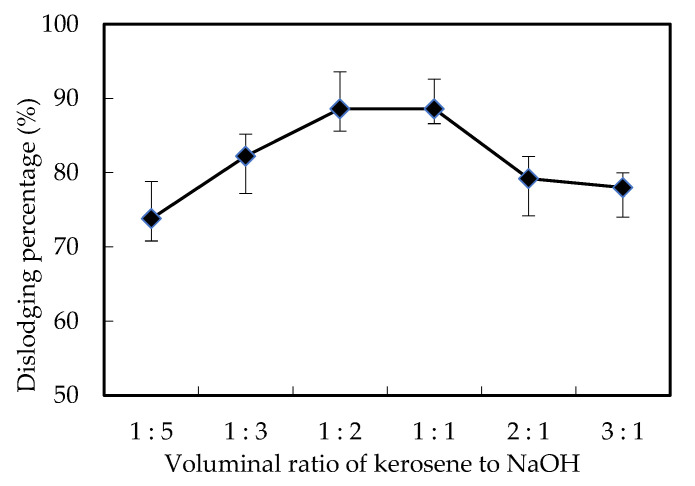
Effects of the voluminal ratio of petroleum to sodium hydroxide on the dislodging efficiency of Cr (VI).

As we described above, the mixed solutions in the supplying cell were a certain voluminal ratio of petroleum with N235/7301 to sodium hydroxide. To investigate the voluminal ratio impacts of the mixed solutions, the ratios of petroleum to sodium hydroxide were adjusted to 1:3, 1:3, 1:2, 1:1, 2:1 and 3:1, which was shown in Figure 4. The result suggested that the dislodging efficiency of Cr (VI) first increased with the voluminal ratio of petroleum to sodium hydroxide when it was lower than 1:1. However, the dislodging efficiency of Cr (VI) decreased sharply when the voluminal ratio of petroleum to sodium hydroxide reached 1:1. This is because when the ratio of sodium hydroxide is low, the acidity difference (dislodging power) on both sides decreases, resulting in a reduction in dislodging efficiency. In addition, some studies have explained that the reduction of sodium hydroxide reduces the stripping efficiency [24].

In our work, the petroleum solution with N235/7301 was ascertained as the membrane solution. By increasing the recycling opportunity of N235/731, the stability of the membrane can be restored to improve the complexation rate and improve the Cr (VI) removal efficiency. When sodium hydroxide was selected as the stripping solution for dislodging Cr (VI), the increase of sodium hydroxide not only increased the stripping rate of [([N235/7301]H)_6_Cr_2_O_7_] decomplexion, but also increased the concentration difference of H^+^ concentration in the feed part and the supply part of the reaction cell. Therefore, the diffusion of [([N235/7301]H)_6_Cr_2_O_7_] and recycled N235/731 was finally increased [23,24]. When the voluminal ratio of the mixed solutions increased, the complexing rate increased while the resolving rate and spreading rate decreased, these reactions equilibrium lead to the maximum dislodging efficiency of Cr (VI) when voluminal ratio of petroleum to sodium hydroxide was 1:1.

**Figure 5:**
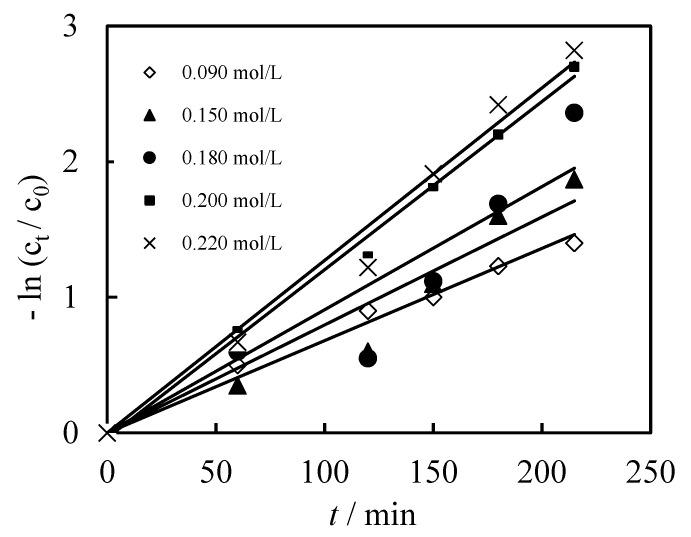
Effects of N235/7301 concentration on the dislodging efficiency of Cr (VI).

In addition, the parameters of N235/7301 also have an important influence on the dislodging of Cr (VI). As can be seen from Equations I and II of Figure 1, the higher the N235/7301 molarity, the higher the probability of forming complex [([N235/7301]H)_6_Cr_2_O_7_] in the membrane system. The higher opportunity of the diffusion and decomplexation of the [([N235/7301]H)_6_Cr_2_O_7_] increased the dislodging efficiency, as shown in Figure 5. Overall, considering the dislodging efficiency and cost of chemical solutions and materials, 0.200 mol/L is the most suitable condition.

We need to pay attention to the selection of the best conditions. We considered the price of materials. As for the price, if the removal efficiency was close under the two conditions, we chose the condition that uses less organic phosphonic acid membrane solution. Because the membrane solution is very expensive, too much membrane solution is not stable. Similarly, if the dislodging efficiency is similar under different acidity or alkalinity conditions, we choose the condition with less acid or alkali. This is because too many acids and bases are unstable.

### 3.3. Impacts of Cr (VI) Adsorption and the Membrane Reuse

**Figure 6:**
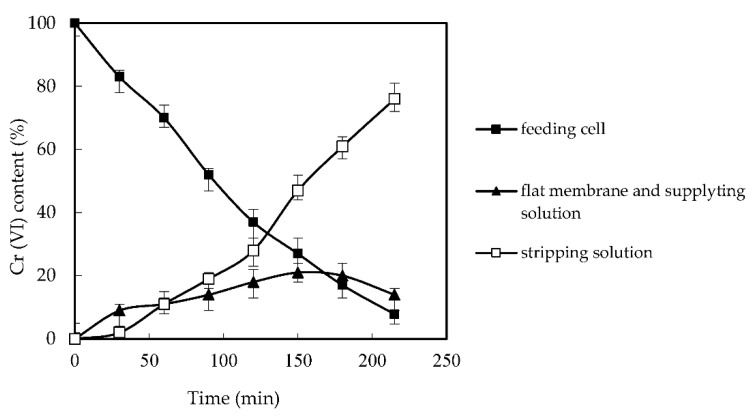
The retention of Cr (VI).

In previous studies using SLM to remove heavy metals, the retention of heavy metal ions on the membrane was observed [12,13]. In this work, Cr (VI) ions have also received attention. The concentration of Cr (VI) on the membrane can be calculated according to the concentration of Cr (VI) in the feed tank and the supply tank. As shown in Figure 6, the adsorption of Cr (VI) on the film increased with the running time. However, with the prolongation of the operation time, the increase rate decreased, which led to a stable Cr (VI) percentage on the membrane when the operation time exceeded 150 min.

**Figure 7:**
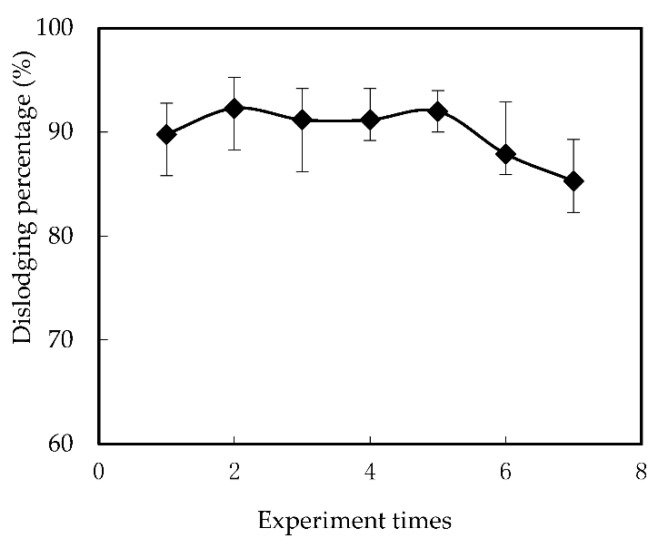
The impacts of reuse time on the dislodging of Cr (VI).

This is because, as the running time increases, the rate of [([N235/7301]H)_6_Cr_2_O_7_] in the interface between the membrane and the supply solution decreased with the increase of Cr (VI) in the supply solution of the reaction part. With the increase of operation time, the equilibrium was reached, and the adsorption of Cr (VI) was no longer increased. There was about 14% Cr (VI) on the membrane surface of the reaction tank. However, this has no negative effect on the supply of the membrane. As shown in Figure 7, when the experiment was repeated five times, the dislodging rate remained above 80%, which verified the practicability and steadiness of FSME. The stability of the membrane and the removal rate of Cr (VI) are also enhanced by the separation of the reaction tank from the feed and supply tanks in the new design. This is the advantage of our newly designed FSME, which avoids the carrier shedding that may be caused by the agitator in the traditional SLM method.

## 4. Conclusions

The running and the influencing factors of our new designed flat supplying membrane equipment (FSME) with mixed N235/7301 dissolved in petroleum and sodium hydroxide solvent for the dislodging of the Cr (VI) from the industrial slops was examined in the laboratory in this work. The results showed that FSME was able to remove Cr (VI) from the simulated industrial slops with high dislodging efficiency, using the mixed petroleum with the carrier of N235/7301 and sodium hydroxide (stripping solution) as the supplying solution. The optimal technology parameters were considered based on the effects of different impact parameters, with consideration of both the dislodging efficiency and the cost of chemical solutions and materials. The result represented the optimal parameters of the FSME, which were: the concentration of carrier (N235/7301) was 0.20 mol/L, the voluminal ratio of petroleum and sodium hydroxide in the supplying cell was 1:1, pH in the feeding cell was 4.00, and the running time was 215 min. Under the optimal conditions, the dislodging rate could reach 93.3%. Although there were many Cr (VI) ions detained on the membrane surface during the operation, the stable retention rate remained below 14% and did not hinder the reuse of the membrane, in which the removal rate of Cr (VI) was higher than 80%.However, the adsorption on the membrane surface should be further solved in the future in order to apply FSME in a wider range.

## Data Availability

Not applicable.
